# Multiomics Provides a New Understanding of the Effect of Temperature Change on the Fermentation Quality of *Ophiocordyceps sinensis*

**DOI:** 10.3390/jof11060403

**Published:** 2025-05-23

**Authors:** Zhengfei Cao, Tao Wang, Hui He, Yuling Li, Xiuzhang Li

**Affiliations:** State Key Laboratory of Plateau Ecology and Agriculture, College of Animal Husbandry and Veterinary Sciences, Qinghai University, Xining 810016, China; c1474477969@163.com (Z.C.); 13085500761@163.com (T.W.); he15226330573@163.com (H.H.)

**Keywords:** temperature stress, metabolite, antioxidant activity, metabolome, transcriptome

## Abstract

*Ophiocordyceps sinensis* is a medicinal fungus with significant nutritional and utilization value. Temperature is a crucial factor influencing its growth, as temperature changes can impact enzyme activity, metabolite content, and gene expression during fungal cultivation. Currently, there are limited reports on the effects of temperature on the quality of fungal fermentation. This study focuses on *O. sinensis* and conducts temperature stress culture experiments. The results indicate that the optimal culture temperature range is between 18 and 23 °C, with extreme temperatures negatively affecting the morphology, growth rate, sporulation, and antioxidant systems of the strains. Further metabolomic and transcriptomic analyses revealed that differentially expressed genes (DEGs) and differentially accumulated metabolites (DAMs) were primarily enriched in four metabolic pathways: linoleic acid metabolism, arginine and proline metabolism, and lysine degradation. Many significantly enriched metabolites across various pathways appear to be predominantly regulated by ribosomal and RNA polymerase genes. Furthermore, we cultured *O. sinensis* mycelium at various temperatures and observed that a significant number of genes and metabolites associated with apoptosis and senescence were expressed at 28 °C. This led to cell damage, excessive energy consumption, and ultimately inhibited mycelial growth. In summary, this study elucidates the response mechanisms of *O. sinensis* to key metabolic pathways under different temperature growth conditions and explores factors contributing to strain degradation.

## 1. Introduce

The Chinese caterpillar fungus, *Ophiocordyceps sinensis* (Berk.) Sung et al. (Ascomycota, Ophiocordycipitaceae), is traditional Chinese medicine, known for its enormous medicinal and commercial value [1,2]. The adenosine, polysaccharides, proteins, and other bioactive compounds abundantly present in it significantly contribute to human health and disease prevention. Research has demonstrated that *O. sinensis* possesses various health benefits, including the ability to lower blood sugar levels, reduce blood lipids, exhibit anti-tumor properties, provide antioxidant effects, combat aging, and promote skin whitening [3,4,5,6,7,8]. Consequently, both the nutritional and medicinal values of *O. sinensis* have garnered recognition among consumers, leading to a year-on-year increase in demand. However, human activities such as farming, grazing, and excessive harvesting have severely compromised its natural habitat. Additionally, environmental challenges such as global warming have further diminished the reserves of wild *O. sinensis*. These pressing supply and demand issues have prompted researchers to explore alternatives to *O. sinensis* to mitigate the resource depletion crisis [9,10,11].

The results showed that the nutrient quality of *O. sinensis* mycelia was affected by the change in culture temperature. When the growth temperature range is between 15 °C and 25 °C, the growth cycle of the fungi is relatively short, allowing for a faster completion of the growth and development process. For instance, the optimal pH for mycelium growth and dry matter weight of *O. sinensis* pupa is 7.0, while the optimal temperature for mycelium growth and dry matter weight is 22 °C [12]. The ideal combination of temperature, pH, and culture time to maximize the yield of cordycepin is 25 °C, 5.5, and 21 days, respectively [13]. When the ambient temperature exceeds 25 °C, the enzyme activity of the fungus significantly decreases and may become deactivated, resulting in changes to the nutrient content of the fungus. Li et al. reported that raising the temperature from 25 °C to 30 °C and 35 °C during the cultivation of the filamentous fungus Aspergillus niger B1-D generally enhanced cellular metabolism and led to the transient accumulation of protein hydroxyl compounds, indicating that the antioxidant function of cells was impaired at high temperatures [14]. Under heat stress, 45.45% of *O. sinensis DnaJ* are differentially expressed, suggesting their involvement in the response to temperature stress and their regulatory role in growth and development [15]. Fungal proteome analysis under high temperature stress revealed that the heat shock protein (HSP) was up-regulated at 20 °C, potentially activating the unfolded protein response (UPR) [16]. Changes in transcriptional responses induced by low temperatures (T < 15 °C) affect several metabolic processes related to nutrient assimilation and energy sources, including amino acid and lipid metabolism, with the lipid biosynthesis pathway being regulated through the transcriptional up-regulation of membrane function [17]. Consequently, the degradation of strains significantly impacts the yield and quality of filamentous fungi. Additionally, degradation causes the accumulation of large amounts of reactive oxygen species (ROS) within the strain. Concurrently, the activity of antioxidant enzymes diminishes, and the levels of exopolysaccharides, cellulase, and amylase become unbalanced, which disrupts the normal physiological processes of the strain and induces oxidative stress reactions [18]. It has been reported that fungi are frequently employed for large-scale industrial production due to their adaptability to various substrates and multifunctional characteristics [19]. Metabolomics is the research object of all metabolites in organisms or cells. The purpose for this field is to reveal the metabolic characteristics and metabolic laws of organisms in different physiological states by studying the changes in metabolites. The changes in these metabolites reflect the metabolic process and physiological state inside the host [20,21]. Zhou et al. determined the matrix and sclertium of wild *O. sinensis* through a wide range of targeted metabolomics and non-targeted metabolomics, and found that niacin and niacinamide metabolism, thiamine metabolism, riboflavin metabolism, glycine, serine and threonine metabolism, and arginine biosynthesis may be key aspects in the differentiation of the matrix and sclertium [22]. Zhong et al. conducted a transcriptomic analysis of pathogenic fungi before and after *O. sinensis* infection, and gene ontology (GO) analysis showed that differentially expressed genes (DEGs) were particularly abundant in aspects related to biological processes and molecular functions [23]. He et al. analyzed changes in Cordyceps sinensis fungus for different fermentation time durations using metabolomics and transcriptomics, revealing that the increase in the contents of DEGs and DAMs promoted the increase in enzyme and non-enzyme substance levels in Cordyceps sinensis, ultimately enhancing the antioxidant capacity of Cordyceps sinensis [24]. However, strain degradation during temperature culture or preservation can result in substantial economic losses [25,26]. Therefore, determining the optimal culture temperature of *O. sinensis* mycelium is not only crucial for the effective preparation of its mycelium but also for large-scale industrial production in the future.

In this study, we established five distinct fungal fermentation temperature treatments at 8 °C, 13 °C, 18 °C, 23 °C, and 28 °C. By analyzing morphological, physiological, and biochemical characteristics, as well as metabolic profiles and gene expression at these varying temperatures, we identified the optimal temperature range for culturing *O. sinensis*. This research provides theoretical support for the industrial-scale production of *O. sinensis* and enhances our understanding of how temperature fluctuations influence the nutrient quality of *O. sinensis* mycelium.

## 2. Materials and Methods

### 2.1. Test Strain and Medium

*O. sinensis* was provided by the *O. sinensis* Laboratory, Academy of Animal Science and Veterinary Science, Qinghai University (Xining City, China), and the strain was stored in the germplasm bank of *O. sinensis* Laboratory of Qinghai Province (no. CX-HN-9).

The solid potato dextrose agar (PDA) medium was composed of 20% potato, 2% glucose, 0.3% peptone, 0.2% potassium dihydrogen phosphate, 0.02% magnesium sulfate, and 1.6% agar, with a pH of 7. This medium was autoclaved at 121 °C for 30 min before use. The liquid PDA medium consisted of the same components: 20% potato, 2% glucose, 0.3% peptone, 0.2% potassium dihydrogen phosphate, and 0.02% magnesium sulfate, with a pH of 7. This medium was dispensed into triangular bottles and also autoclaved at 121 °C for 30 min before use.

### 2.2. Treatment and Collection of Mycelia

The culture of solid mycelia was initiated by using a hole punch with a diameter of 0.25 cm to extract an equal amount of mycelia from the center of a solid culture dish. This mycelial sample was then inoculated at the center of a PDA solid culture medium and incubated at temperatures of 8 °C, 13 °C, 18 °C, 23 °C, and 28 °C, respectively.

Culture of liquid mycelium: An equal amount of strain was extracted from the previous operation, homogenized using an inoculating shovel, and subsequently inoculated into PDA liquid medium. The cultures were maintained under five different temperature conditions, with a shaking speed of 135 rpm. The culture was deemed complete when 80% of the mycelium had proliferated within the liquid medium at the specified temperature. Following centrifugation at 4 °C, the supernatant was discarded (10,000 g), yielding the cultured mycelium, which was then stored in a cryogenic freezer at −80 °C.

### 2.3. Growth Rate, Apparent Morphology, SEM Analysis, Dry and Fresh Weight, and Conidial Weight Were Measured

Scanning electron microscopy (SEM) analysis was conducted to observe the mycelial growth morphology at various temperatures. Mycelia were collected at 8 °C, 13 °C, 18 °C, 23 °C, and 28 °C, and fixed in 2.5% glutaraldehyde at 4 °C for 12 h. The samples were rinsed three times with a 0.1 mol/L phosphate buffer, with each rinse lasting 15 min. They were then dehydrated using a series of ethanol solutions (30%, 50%, 70%, 90%, and 95%), followed by two rounds of dehydration in 100% ethanol, each lasting 20 min. The samples were subsequently treated with a mixture of ethanol and isoamyl acetate (in a 1:1 volume ratio) for 30 min, followed by treatment with isoamyl acetate alone for 2 h. Finally, the samples were placed in a freeze dryer for 15 h. The dried fungi were affixed to the sample table for gold-spraying treatment and observed under scanning electron microscopy to assess the thickness, curvature, branching, and surface characteristics of the mycelia at each temperature gradient, after which photographs were taken and recorded.

Three biological replicates were established for each measurement. After ten days of culture, the colony diameter was measured using a ruler and the cross-crossing method every two days. The growth of colonies at different temperatures was observed, and their morphology was described in terms of colony growth rate, mycelium status on the colony surface, mycelium texture, colony color, and colony shape. Photographs were taken and recorded for documentation. At the end of 22 days of culture, the entire mycelium was removed along with the medium, which was subsequently washed off with hot sterile water. The residual water on the colony surface was absorbed using filter paper (Hangzhou City, China), and the fresh weight of the colony was determined using an electronic balance. Finally, the colonies were placed in a constant temperature drying box set at 55 °C, where they were weighed every 30 min until the mass stabilized over three consecutive measurements. The average of these three measurements was recorded as the dry weight of the colonies. To prepare a spore suspension, 5 mL of sterile water was added to the colony plate, and the colony surface was gently brushed to dislodge the conidia. The mycelium, media, and other impurities were filtered out using gauze rinsed with sterile water. A 0.05% Tween-80 solution was used to create a spore suspension with a fixed volume of 10 mL, which was thoroughly mixed using a vortex oscillator (Shanghai City, China). A hemocytometer was employed to count the number of spores produced by a single colony. The results were subsequently calculated using the formula provided below.

Conidial number (each/mL) = total number of small square spores (80)/80 × 400 × 10^4^ × dilution.

### 2.4. Determination of Antioxidant Enzymes

This study measured several antioxidant enzymes, specifically catalase (CAT), superoxide dismutase (SOD), and peroxidase (POD). The measurement methods strictly adhered to the protocols established by Suzhou Keming Biotechnology Co., Ltd. (Suzhou City, China). The assays were performed following the detailed instructions provided in the corresponding kit.

### 2.5. Extraction of Metabolites

The extraction method for metabolites was refined based on our laboratory’s research findings and relevant published studies [27,28]. Initially, 100 mg of the sample was placed in a 2 mL centrifuge tube, to which 6 mm diameter glass beads were added, and the mixture was ground at a low temperature (4 °C). Subsequently, 80 mg of the pulverized sample was combined with 1000 μL of extractant (composed of 40% methanol, 40% acetonitrile, and 20% water) and swirled for 30 s. A tissue grinder (JXFSTPRP-CLN-24, Shanghai, China) was employed for mixing, with parameters set to −10 °C, 55 Hz, for 2 min. The mixture was then subjected to ultrasonic extraction using a cell comminuter (JC-12L, Guangdong, China) at 4 °C, applying a force of 13,000 g for 10 min, after which it was removed. The liquid mixture was centrifuged at 12,000 rpm for 10 min at 4 °C, and the supernatant was collected, concentrated, and dried. Finally, the supernatant was filtered through a 0.22 μm membrane and injected into the test bottle.

### 2.6. LC-MS/MS

The metabolomics analysis was performed using the UPLC-ESI-Q-orbital well MS system (UHPLC, Shimazu Nexera X2 LC-30AD, Shimazu Corporation, Kyoto, Japan) in conjunction with Q-Exactive Plus (Thermo Scientific, San Jose, CA, USA). For liquid chromatography (LC) separation, samples were analyzed using the ACQUITY UPLC and HSS T3 column (2.1 × 100 mm, 1.8 μm) (Waters, Milford, MA, USA). During the preparation of the experiment, we mixed all samples in equal amounts to obtain the quality control (QC) samples. During data preprocessing, we adopted QC sample-based normalization for substance quantification: support vector regression correction based on QC samples to eliminate systematic errors. Subsequently, substances with a coefficient of variance (CV) of less than 30% in the QC samples were retained for further analysis. The parameters were set as follows: flow rate of 0.3 mL/min, column temperature of 40 °C, and sample size of 2 μL. The mobile phase consisted of A (0.1% formic acid in water) and B (0.1% formic acid in acetonitrile). Linear gradient elution was conducted on the samples in both negative (NEG) and positive (POS) modes, with the following gradient: 0–1 min at 8% B; 1–8 min from 8% to 98% B; 8–10 min at 98% B; 10–10.1 min from 98% to 8% B; and 10.1–12 min at 8% B. The parameters for the mass spectrometer were configured as follows: the positive ion spray voltage was set to 3.50 kV, the negative ion spray voltage was set to −2.50 kV, the sheath gas flow was maintained at 40 arb, and the auxiliary gas flow was maintained at 10 arb. The capillary temperature was set to 325 °C, with first-level full scanning conducted at a resolution of 60,000. The first-level ion scanning range was set from 100 to 1000 *m*/*z*, and second-level fragmentation was performed using HCD with a collision energy of 30%. The second-level resolution was set to 15,000, and the first four ions were fragmented following signal acquisition, with unnecessary MS/MS data excluded via dynamic exclusion. MS-DIAL was employed to conduct peak alignment, retention time correction, and peak area extraction on the original mass spectrometry data. Metabolites were identified based on mass (mass tolerance < 10 ppm) and MS/MS data (mass tolerance < 0.02 Da), which were accurately matched with public databases such as the Human Metabolome Database (HMDB), Mass Bank, and our self-constructed metabolite standard library. Among the extracted ion features, only variables with more than 50% non-zero measurements in at least one dataset were retained.

### 2.7. Multivariate Statistical Analysis

The R software (v4.3.1) package was utilized for conducting principal component analysis (PCA), partial least squares discriminant analysis (PLS-DA), and orthogonal partial least squares discriminant analysis (OPLS-DA) for dimensionality reduction in the sample data. The model’s overfitting was assessed using the permutation test method. R^2^X and R^2^Y denote the proportion of variance explained by the model for the X and Y matrices, respectively, while Q^2^ indicates the model’s predictive capability. Values closer to 1 indicate a better fit of the model. The *p* value was computed based on statistical tests, the variable importance in projection (VIP) was derived from the OPLS-DA dimensionality reduction method, and the fold change between groups was calculated using log_2_. The influence and interpretative capacity of each metabolite on sample classification and discrimination were evaluated, facilitating the selection of metabolites. Metabolites with a *p* value 1 were deemed to exhibit statistically significant differences, and the identified differential metabolites were subjected to cluster analysis using the R package. KEGG pathway analysis was conducted on the differential metabolite data using the KEGG database (http://www.kegg.jp, accessed on 9 October 2024). The Fisher precision test was employed for KEGG enrichment analysis, with false discovery rate (FDR) correction applied for multiple comparisons. The enriched KEGG pathways were considered statistically significant at a *p* < 0.05 level.

### 2.8. RNASeq Process

Total RNA was enriched for mRNA with a polyA structure using Oligo (dT) magnetic beads. The RNA was fragmented into approximately 300 bp pieces through ion interruption, and these fragments were selected for further analysis. Using RNA as a template, the first strand of cDNA was synthesized with 6-base random primers and reverse transcriptase, followed by the synthesis of the second strand of cDNA using the first strand as a template. PCR amplification was employed to enrich the library fragments, resulting in a library size of 450 bp. The library was assessed using the Agilent 2100 Bioanalyzer (100 nm × 2.1 nm, 1.7 μm, Beijing City, China) to determine both the total and effective concentrations. Based on the effective concentration and the required data amount, libraries containing different index sequences were mixed proportionally and diluted to 2 nM. An alkaline denaturation process was then applied to form a single-stranded library. Paired-end sequencing was conducted on the sample library after RNA extraction, purification, and library construction, utilizing Illumina’s Next-Generation sequencing technology. Reads that were spliced, shorter than 50 bp, or had an average quality below Q20 were discarded. The remaining high-quality sequences were assembled from scratch to obtain transcript sequences. The transcripts were clustered, and the longest transcript was designated as Unigene for subsequent Gene Ontology (GO) and Kyoto Encyclopedia of Genes and Genomes (KEGG) annotation. Concurrently, the filtered sequences were compared to Unigene to obtain the Reads Count for each Unigene, followed by differential expression and enrichment analysis. Finally, the differentially expressed genes were analyzed to elucidate changes in gene expression under various conditions and to uncover potential biological significance.

### 2.9. Real-Time Quantitative Fluorescence (qRT-PCR) Verification

To validate the RNA-seq data, we selected 15 differentially expressed genes (DEGs) for quantitative reverse transcription polymerase chain reaction (qRT-PCR) validation. Primers were designed using the Primer Quest Tool (Oligo v7.56), with a preference for those located near the 3′ end of the genes. The reaction mixture consisted of 5 µL of 2× PerfectStart^®^ Green (Beijing Quanshijin Biotechnology Co., Ltd., Beijing City, China) qPCR Super Mix, 0.2 µL of forward primer (10 µM), 0.2 µL of reverse primer (10 µM), and 1 µL of cDNA. The cycling conditions were set at 94 °C for 30 s, followed by 94 °C for 5 s and 60 °C for 30 s. The relative expression levels of the target genes were analyzed using the 2^−ΔΔCt^ method, with three biological replicates for each treatment.

### 2.10. Data Processing and Analysis

Microsoft Office Excel 2019 was utilized to organize the data, while IBM SPSS Statistics 26.0 software was employed to perform one-way ANOVA on the data that satisfied the assumptions of normal distribution and homogeneity of variance. The Waller–Duncan test was then applied to analyze the significance of differences among multiple sample groups. The data analysis in this work was supported by Allbioknow Biotechnology Co., Ltd. (Chongqing, China).

## 3. Result

### 3.1. Growth Rate, Apparent Morphology, Dry and Fresh Weight, Conidial Mass, and SEM Analysis of O. sinensis at Different Temperatures

**Figure 1:**
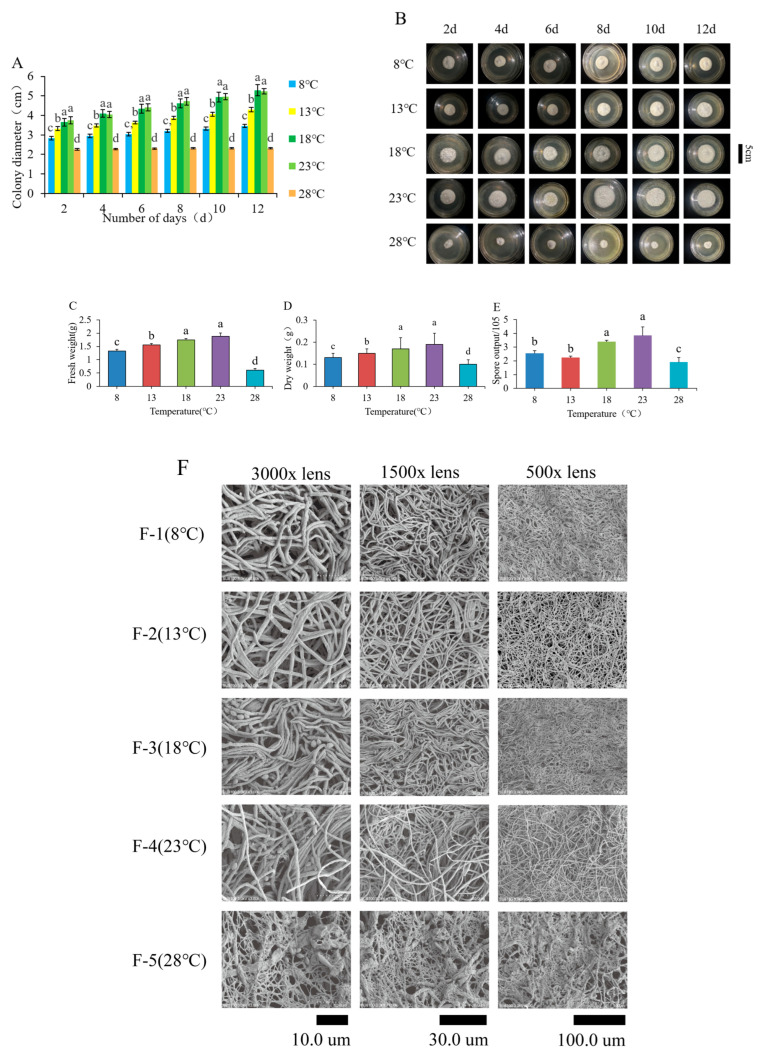
The (**A**) growth rate, (**B**) apparent morphology, (**C**) fresh weight, (**D**) dry weight, (**E**) conidial count, and (**F**) SEM images of the strains at different temperatures. Different lowercase letters indicated significant differences (*p* < 0.05).

The growth of fungi was observed at temperatures of 8 °C, 13 °C, 18 °C, 23 °C, and 28 °C. The results indicated that *O. sinensis* exhibited the fastest growth at 18 °C and 23 °C, with no significant difference between these temperatures (*p* > 0.05). This was followed by growth at 13 °C and 8 °C, while growth at 28 °C was very slow or completely halted (*p* < 0.05). These findings suggest that the optimal temperature range for the growth of *O. sinensis* is between 18 °C and 23 °C. The surface morphology of the mycelia varied with different temperature treatments. As illustrated in Table 1 and Figure 1A,B, mycelia grown at 8 °C and 13 °C appeared fluffy and raised, whereas those at 18 °C and 23 °C exhibited a smooth and fluffy surface. In contrast, the mycelia at 28 °C appeared raised. The texture, margin, and color of the colonies were consistent across all temperatures: flocculent, ciliated, and white. The colonies at 8 °C and 13 °C were circular and quasi-circular, while those at 18 °C and 23 °C were circular and demonstrated the best growth. The colonies at 28 °C were quasi-circular. Significant differences were observed in colony shape. Notably, the colonies at 18 °C and 23 °C showed similar growth patterns, indicating optimal conditions, followed by those at 13 °C and 8 °C. At 28 °C, colony growth was inhibited, and yellow precipitate was observed at the edges. The growth at 13 °C surpassed that at 8 °C, while growth at 28 °C was the least favorable. Over time, at 8 °C, 13 °C, 18 °C, and 23 °C, many airborne mycelia were observed growing on the colony surface, indicating the continuous formation of new branches. At 28 °C, the colony exhibited tough characteristics, with numerous uplifted folds and extremely slow growth. This suggests that temperature significantly influences the growth of *O. sinensis*, with a sharp decrease in the number of primordia formed at higher temperatures.

The growth patterns of mycelium varied with different temperature treatments, highlighting the pronounced effects of temperature on growth. The typical characteristics of mycelia at 8 °C included uniform thickness, minimal branching, random arrangement, and a predominant intertwining distribution (Figure 1F(F-1)).

**Figure 2:**
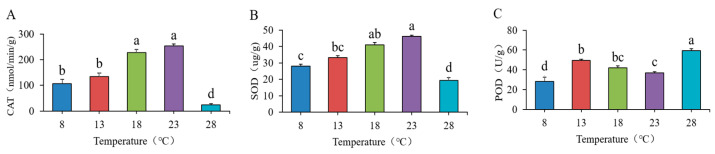
Changes in (**A**) CAT, (**B**) SOD, and (**C**) POD in strains at different temperatures. Different lowercase letters indicate significant differences (*p* < 0.05).

At 13 °C: the curvature of the mycelia is markedly pronounced, with no branching observed, and overlapping growth is prominent. The growth appears relatively organized, and the arrangement is regular (Figure 1F(F-2)).

**Figure 3:**
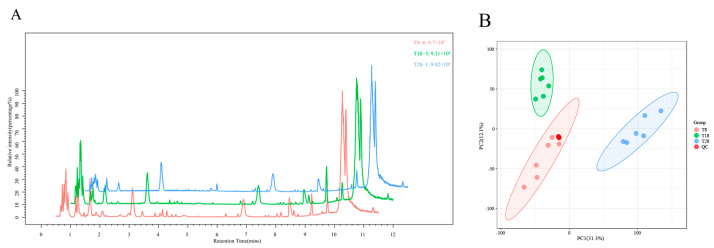
(**A**) Mass spectrum base peak diagram among the three groups of samples; (**B**) PCA scatter score diagram.

At 18 °C: the surface exhibits distinct folds, a relatively loose arrangement, no branching, and a certain degree of curvature. A significant number of conidia are produced on the surface of the mycelium, the majority of which are kidney-shaped, while a minority are oval in shape. These conidia either fall off or adhere to the mycelium after their formation (Figure 1F(F-3)).

**Figure 4:**
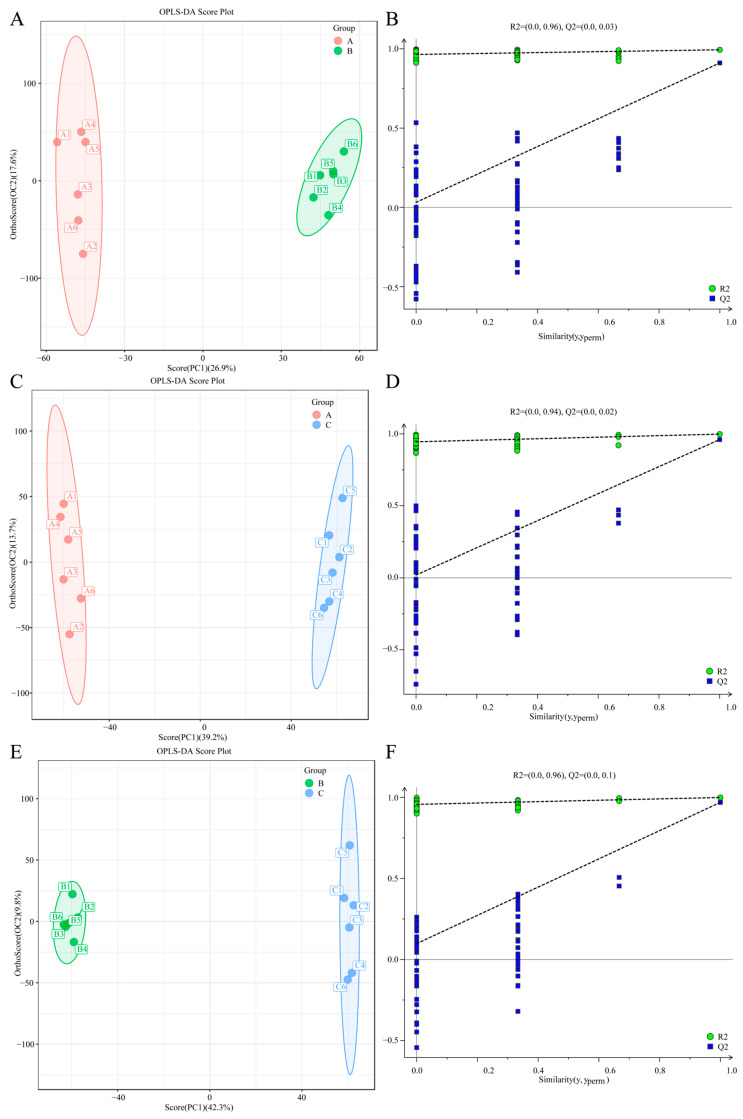
(**A**–**F**) represent OPLS-DA score plots and permutation test plots of T8 vs. T18, T8 vs. T28 and T28 vs. T18, respectively.

At 23 °C: non-uniform thickness, absence of branches, a close and relatively distinct arrangement, and a significant presence of conidia (Figure 1F(F-4)).

**Figure 5:**
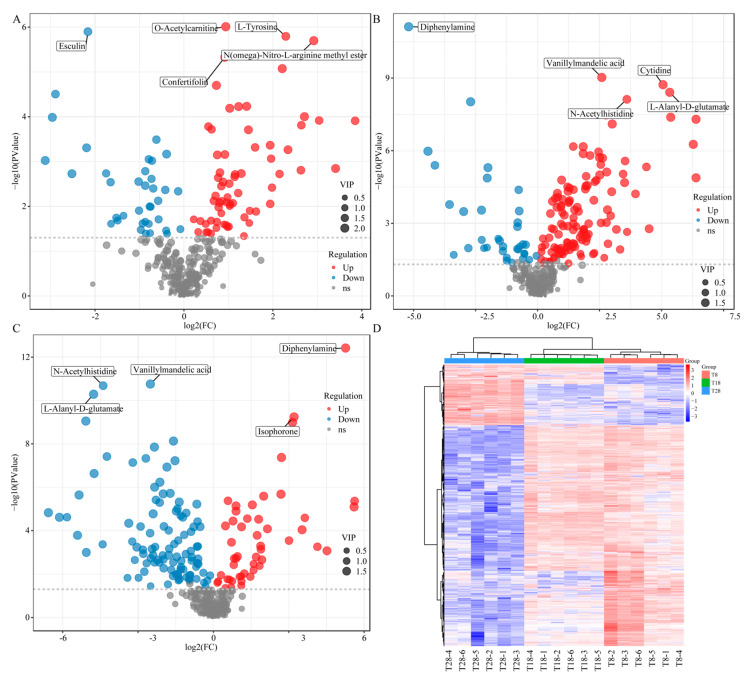
Volcanic plots of differential metabolites: (**A**) T8 vs. T18, (**B**) T8 vs. T28, and (**C**) T28 vs. T18. (**D**) T8 vs. T18 vs. T28 hierarchical cluster map.

The typical characteristics of mycelia at 28 °C include non-uniform thickness, close arrangement, and a limited presence of conidia. The growth of mycelia is organized in a network formation, with noticeable interlacing, overlapping, and fusion phenomena, which may be attributed to the effects of high-temperature growth (Figure 1F(F-5)).

With an increase in temperature, both the fresh weight and dry weight of the colonies exhibited significant increases between 8 °C and 23 °C, mirroring the trend observed in the mycelial growth rate. Notably, the fresh and dry weights of the colonies in the 23 °C treatment group reached their peak, while those in the 28 °C treatment group were the lowest, exhibiting a significant difference (*p* < 0.05). There were no significant differences between the 8 °C and 13 °C treatment groups, the 13 °C and 18 °C treatment groups, or the 18 °C and 23 °C treatment groups (*p* > 0.05) (Figure 1C,D). These results further demonstrate that temperature changes have a profound inhibitory effect on the growth of *O. sinensis*, suggesting that the colony may experience rapid growth or even cease growth beyond a certain temperature threshold.

The results indicated that mycelium sporulation in the 23 °C treatment group reached the highest level, followed closely by the 18 °C treatment group, with only a minor difference between them. The colonies growing at 8 °C and 13 °C exhibited no significant difference (*p* > 0.05). The lowest conidial concentration was observed in the 28 °C treatment group, which was significantly different from that in the 23 °C treatment group (*p* < 0.05). The conidia content of *O. sinensis* was ranked from highest to lowest as follows: 23 °C > 18 °C > 8 °C > 13 °C > 28 °C. The respective conidial concentrations were 3.85 ± 0.63 × 10^5^, 3.40 ± 0.10 × 10^5^, 2.55 ± 0.19 × 10^5^, 2.25 ± 0.09 × 10^5^, and 1.90 ± 0.33 × 10^5^.

### 3.2. Evaluation of Antioxidant Enzymes in O. sinensis

As the temperature in the treatment group increased, the activity of catalase (CAT) exhibited a significant increase, with the highest CAT content recorded in the 23 °C treatment group at (254.03 ± 7.58 nmol/min/g). Conversely, at 28 °C, CAT activity sharply declined to (24.37 ± 4.82 nmol/min/g) (*p* < 0.05) (Figure 2A). Superoxide dismutase (SOD) content increased gradually with rising temperatures but also experienced a sharp decrease at 28 °C (*p* < 0.05). The maximum SOD content was measured at 41.09 ± 1.33 µg/g and 46.2 ± 0.58 µg/g at 18 °C and 23 °C, respectively (Figure 2B). Peroxidase (POD) levels initially increased before declining with further temperature increases, reaching its peak at 28 °C, which was significantly higher than the levels observed for other temperature treatments (*p* < 0.05) (Figure 2C). These results indicate that culture temperature significantly influences the antioxidant capacity of *O. sinensis*, displaying distinct trends. Low-temperature environments promote the synthesis of CAT and SOD, while high-temperature environments yield the highest POD content. At lower temperatures, the metabolic processes of the enzymes slow down, which also affects their activity, suggesting that the strain possesses robust antioxidant capacity and environmental adaptability.

### 3.3. Analysis of Metabolic Profile of O. sinensis

#### 3.3.1. Metabolic Profile Characteristics of *O. sinensis* at Different Temperatures

Given that the strains were subjected to treatments at varying temperatures, notable differences emerged in morphology, as well as physiological and biochemical indices. Consequently, we selected the low-temperature treatment group, T8; the moderate-temperature treatment group, T18; and the high-temperature treatment group, T28, for analysis to elucidate the changes in metabolite content across the three temperature conditions. MassLynx was utilized to collect and process data for the total peak normalization of peak area. By analyzing the high ion intensity peaks in the chromatographic base peak diagram, we gained insights into the compositional information of the main components present in the sample. The results indicated that the characteristic peak intensity and retention time of the chromatograms among the samples within each group were nearly consistent, suggesting that the variation due to instrumental errors during the analysis was minimal and that the data quality was reliable (Figure 3A). Principal component analysis (PCA) was employed to cluster each sample based on expression levels, facilitating an understanding of the variations both between and within sample groups as a whole, and allowing for the observation of overall distribution trends among the samples. A closer distance between samples indicates a higher similarity. The analysis revealed that the contribution rate of the first principal component was 33.1%, while that of the second principal component was 12.6%. This suggests significant differences among the temperature treatment groups, with minimal differences observed within the groups. Additionally, the QC samples tended to aggregate, indicating good repeatability within the 95% confidence interval (Figure 3B).

#### 3.3.2. Multivariate Statistical Analysis of Different Samples

Before conducting multivariate statistical analysis, the data underwent log2 transformation followed by standardization (scaling). In this analysis, an ‘adaptive conversion’ method was employed to enhance interpretability. The R language’s ropls package was utilized for multivariate statistical analysis, which includes principal component analysis (PCA), orthogonal partial least squares discriminant analysis (OPLS-DA), and S-Plot analysis for differential multiple analysis. Orthogonal partial least squares discriminant analysis (OPLS-DA) is a supervised statistical method for discriminant analysis that effectively differentiates differential abundance metabolites (DAMs) among various samples. By employing pairwise comparisons using the OPLS-DA model, OPLS-DA score plots can be generated. Similarly to the results obtained from principal component analysis (PCA), a distinct separation among the three sample groups is evident (Figure 4A,C,E). Additionally, the OPLS-DA model, assessed through 100 permutation tests, demonstrated the following results: T8 vs. T18 (R^2^X(cum) = 0.445, R^2^Y(cum) = 0.994, Q^2^(cum) = 0.911); T8 vs. T28 (R^2^X(cum) = 0.529, R^2^Y(cum) = 0.998, Q^2^(cum) = 0.960); and T28 vs. T18 (R^2^X(cum) = 0.521, R^2^Y(cum) = 0.999, Q^2^(cum) = 0.969) (Figure 4B,D,F). In all pairwise comparison groups, R^2^X exceeded 0.4, and the values for R^2^Y and Q^2^ were consistently high, indicating that the OPLS-DA model possesses strong predictability and reliability, thus effectively screening for differential metabolites.

#### 3.3.3. DAMs Identification and Pathway Enrichment Analysis

By comparing the metabolites between T8 vs. T18, T8 vs. T28, and T28 vs. T18, differentially accumulated metabolites (DAMs) were screened with a variable importance in projection (VIP) threshold of ≥1. The results indicated that 103 DAMs (66 up-regulated and 37 down-regulated) were identified in the T8 vs. T18 comparison, including metabolites such as L-Threonine and Catechol. In the T8 vs. T28 comparison, a total of 162 DAMs (127 up-regulated and 35 down-regulated) were identified, including L-Prolinamide and Taurine. Furthermore, the T28 vs. T18 comparison revealed 153 DAMs (50 up-regulated and 103 down-regulated), which included Creatine and Acetylphosphate (Figure 5A–C). The analysis of the differences among the groups indicated that significantly different substances could inhibit mycelial growth and spore germination, possibly due to alterations in cell membrane integrity induced by temperature variations. When organisms encounter adverse conditions such as high temperatures and drought, the generation and elimination of free radicals within cells may become compromised, leading to membrane degradation and peroxidation. The hierarchical clustering analysis revealed that the accumulation patterns of DAMs across all temperature treatment groups could be categorized into two distinct types: the first type exhibited an up-regulated trend in the T28-treated strains, while the second type showed an up-regulated trend in the T8- and T18-treated strains. This finding suggests significant variability in DAMs among different temperature treatment groups (Figure 5D).

**Figure 6:**
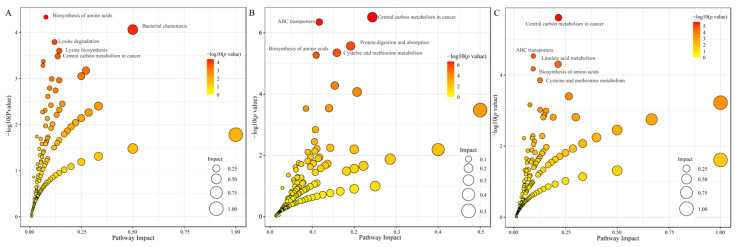
Bubble diagrams of top 20 KEGG enrichment pathways: (**A**) T8 vs. T18, (**B**) T8 vs. T28, (**C**) T28 vs. T18.

Through the KEGG enrichment pathway analysis, we identified the key metabolic pathways of strains cultured at different temperatures. The top 20 enriched metabolic pathways were analyzed, revealing that in the two comparison groups of T8 vs. T18 and T8 vs. T28, three metabolic pathways—lysine degradation, lysine biosynthesis, and protein digestion and absorption—were significantly enriched. However, in the comparison of T28 vs. T18, two metabolic pathways—linoleic acid metabolism and cysteine and methionine metabolism—were significantly enriched (Figure 6A–C).

### 3.4. Transcriptome Analysis

#### 3.4.1. Transcription Profile Analysis

To investigate the changes in gene expression among *O. sinensis* induced by temperature, transcriptome sequencing was conducted on samples from T8, T18, and T28, with three biological replicates for each. A total of 41,638,054 clean reads and 62,633,656,824 clean bases were obtained after eliminating low-quality reads, resulting in a potential overall sequencing error rate of less than 0.005%. The distribution of Q20 bases ranged from 97.33% to 97.43%, while Q30 bases ranged from 95.22% to 95.45%. The average GC content was 54.58%. These results indicate that the transcriptome sequencing was of high quality and met the requirements for subsequent analyses. The quality assessment of the sequencing data is presented in Table 2.

**Figure 7:**
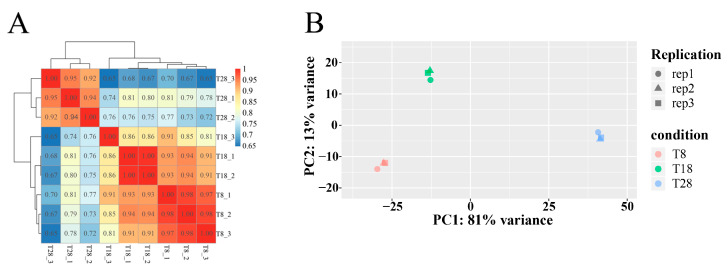
(**A**) Sample correlation test. The left and upper sides are sample clustering, the right and lower sides are sample names, and squares of different colors represent the level of correlation between two samples. (**B**) Sample PCA. The horizontal coordinate is the first principal component, and the vertical coordinate is the second principal component. Different shapes in the figure represent different samples, and different colors represent different groupings.

The correlation of gene expression levels between samples serves as a crucial metric for assessing the reliability of the experiment and the appropriateness of sample selection. In this study, the Pearson correlation coefficient was utilized to evaluate the gene expression correlation between samples; a coefficient closer to 1 indicates a higher similarity in expression patterns. Within the test group, a strong correlation coefficient was observed between the T8 and T18 treatment groups, whereas the correlation coefficient between the T28 treatment group and both the T8 and T18 treatment groups was low, likely due to high-temperature stress. Notably, the correlation coefficients within each sample group exceeded 0.86, indicating a strong correlation and suggesting that the biological replicates exhibit good repeatability (Figure 7A). A principal component analysis (PCA) was conducted on each sample based on expression levels, allowing for the assessment of sample similarity through distance metrics. The internal structure of the multivariable data were elucidated by the first principal component (PC1) and the second principal component (PC2). The results revealed that the first principal component accounted for 81% of the variance, while the second principal component accounted for 13%, indicating significant differences among the temperature treatment groups, with minimal differences within the groups. Both the correlation analysis and PCA demonstrate that the sample correlations among the temperature treatment groups are robust, fulfilling the requirements for subsequent analyses (Figure 7B).

#### 3.4.2. Differentially Expressed Genes

**Figure 8:**
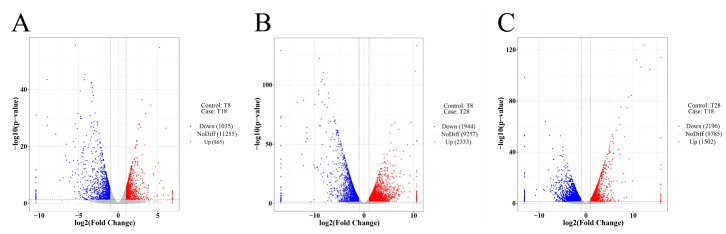
Differential expression volcano plots: (**A**) T8 vs. T18, (**B**) T8 vs. T28, and (**C**) T28 vs. T18.

**Figure 9:**
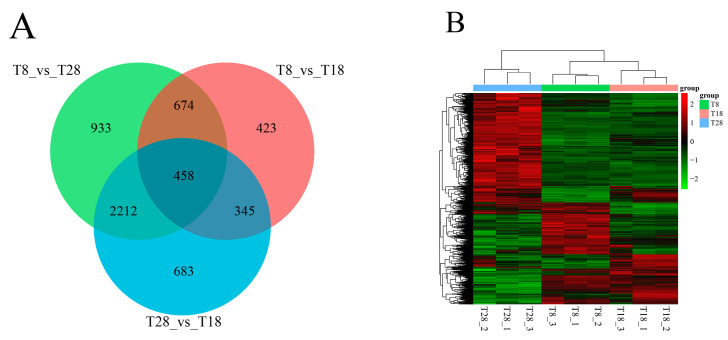
(**A**) Comparison of common and specific differentially expressed genes among different groups; (**B**) cluster analysis of differentially expressed genes.

We analyzed the gene expression profiles of three strains subjected to different temperature treatments, applying a differential expression criterion of |log2FC| > 1 and a significance threshold of *p*-value < 0.05. The results indicated that the number of DEGs was notably similar across the treatment groups. Specifically, a total of 1900 DEGs (865 up-regulated and 1035 down-regulated) were identified in the comparison of T8 vs. T18, while 4277 DEGs (2333 up-regulated and 1944 down-regulated) were found in T8 vs. T28. In the T28 vs. T18 comparison, 3698 DEGs (1502 up-regulated and 2196 down-regulated) were identified (Figure 8A–C). Venn diagram was constructed to illustrate the common and unique DEGs among the different groups. The analysis revealed that the maximum number of unique DEGs was 933 in the T8 vs. T28 group, followed by 683 in the T28 vs. T18 group, and at least 423 in the T8 vs. T18 group. Among these, there were 458 DEGs common to all three comparison groups, 2670 DEGs unique to the T8 vs. T28 and T28 vs. T18 comparisons, and 1132 DEGs unique to the T8 vs. T28 and T8 vs. T18 comparisons. The T8 vs. T18 and T28 vs. T18 groups exhibited a minimum of 803 DEGs (Figure 9A). A cluster analysis of the differentially expressed genes demonstrated that the samples distinctly grouped into two categories: the T28 group and the combined T8 and T18 groups. The low correlation between these two categories suggests that high-temperature stress may alter the original gene expression patterns of *O. sinensis*. When exposed to high temperatures, *O. sinensis* must promptly adjust its gene expression to adapt to changes in ambient temperature. In the T8 and T18 treatment groups, low temperature had an impact on the growth rate of the strains, but compared to T18, they also exhibited favorable growth conditions. Therefore, the differential expression patterns between these two groups were minimal, indicating a high level of correlation. Therefore, the gene expression pattern during this period can be preliminarily identified as a response phase for the organism to rapidly adapt to temperature stress (Figure 9B).

#### 3.4.3. GO Channel Analysis and KEGG Channel Analysis

**Figure 10:**
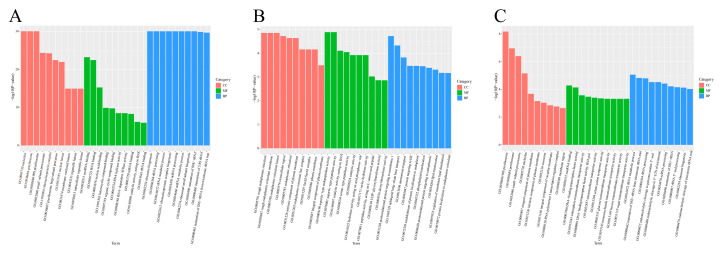
GO enrichment analysis of (**A**) T8 vs. T18, (**B**) T8 vs. T28, and (**C**) T28 vs. T18.

According to the enrichment analysis, Gene Ontology (GO) enrichment analysis was conducted based on molecular function (MF), biological process (BP), and cellular component (CC). The results indicated that 4206 GO terms were enriched in the comparison of T8 vs. T18, 6190 GO terms in T8 vs. T28, and 5850 GO terms in T28 vs. T18 (Figure 10A–C). In the T8 vs. T18 comparison, the cellular components primarily involve the nucleolus (GO: 0005730), pre-ribosome (GO: 0030684), and 90S ribosome (GO: 0030686). The molecular functions are predominantly associated with small RNA binding (GO: 0030515) and ribonucleic acid binding (GO: 0003723). The biological processes mainly pertain to ribosome biogenesis (GO: 0042254), ribosome processing (GO: 0006346), and metabolic processes (GO: 0016072). In the T8 vs. T28 comparison, the cellular components chiefly involve extracellular regions (GO: 0005576), the intrinsic components of the plasma membrane (GO: 0031226), and the plasma membrane (GO: 0005886). The significantly enriched GO terms in the T28 vs. T18 comparison were primarily related to membrane components (GO: 0005887), cotransport activity (GO: 0015293), and solute cation symporter activity (GO: 0015294).

**Figure 11:**
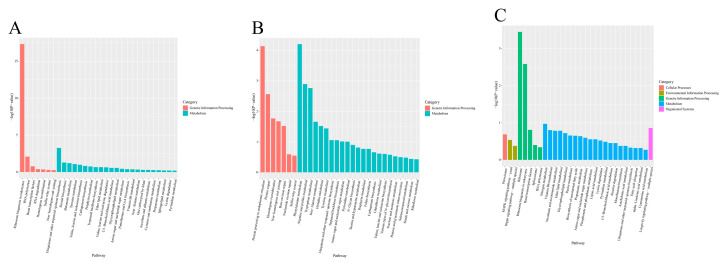
Annotation diagrams of KEGG pathway enrichment analyses: (**A**) T8 vs. T18, (**B**) T8 vs. T28, (**C**) T28 vs. T18.

**Figure 12:**
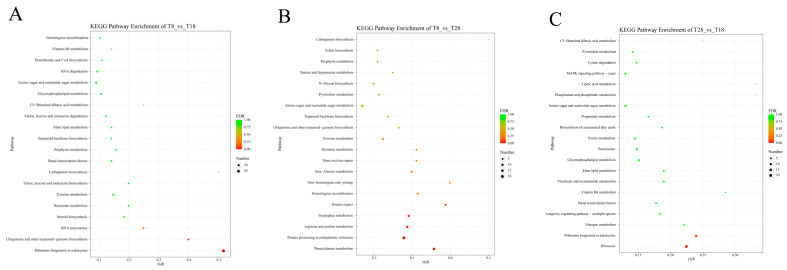
Bubble maps of KEGG enrichment analyses (top 20, *p* < 0.05): (**A**) T8 vs. T18, (**B**) T8 vs. T28, (**C**) T28 vs. T18.

In addition, we selected the top 20 pathways with a *p*-value < 0.05 for analysis to understand the effect of temperature change on the enrichment of differentially expressed genes (DEGs) pathways. The results indicate that DEGs in the comparison of T8 vs. T18 are highly enriched in the KEGG pathways related to ribosome biogenesis in eukaryotes, ubiquinone, other terpenoid–quinone biosyntheses, and RNA polymerase. In the comparison of T8 vs. T28, DEGs are primarily involved in the KEGG pathways of protein export, tryptophan metabolism, and arginine and proline metabolism, with significant enrichment also observed in metabolism, protein processing in the endoplasmic reticulum, and phenylalanine metabolism. The KEGG pathway analysis of DEGs in T28 vs. T18 shows significant enrichment in ribosome and ribosome biogenesis in eukaryotes (Figure 11A–C). Based on the KEGG enrichment results, the top 20 significantly enriched and up-regulated KEGG pathways were further analyzed through bubble maps. The findings demonstrate that these significantly enriched KEGG pathways were notably up-regulated across the three different comparison groups. This suggests that when temperature stress is applied to *O. sinensis*, there may be osmoregulatory substances, primarily ribose and enzymes, present in its strain, which facilitate the transport of related substances through mycelial connections within *O. sinensis* (Figure 12A–C).

### 3.5. Combined Analysis of the Transcriptome and Metabolome

#### 3.5.1. Comprehensive Analysis of DAMs and DEGs

**Figure 13:**
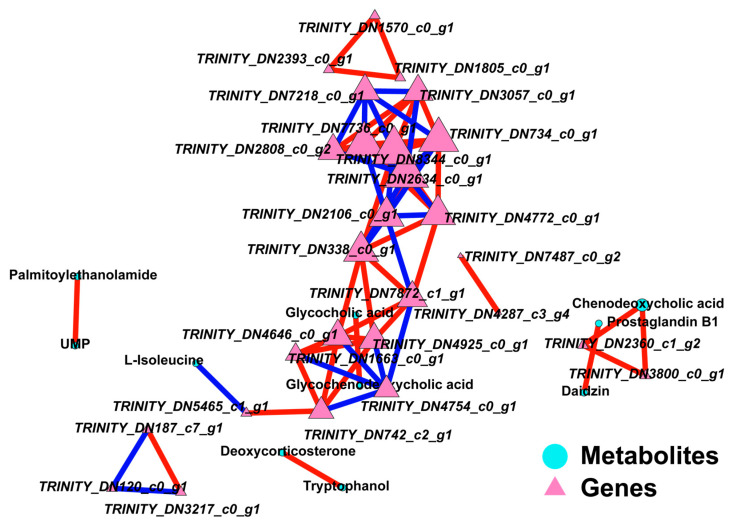
Pathway maps of DAMs and DEGs from different comparison groups. Among them, the blue line represents a positive correlation, and the red line represents a negative correlation.

To clarify the correlation between differentially abundant metabolites (DAMs) and differentially expressed genes (DEGs) across the three treatment groups, we conducted a joint analysis. The results indicated that the correlations between pentanamide, N-Acetylhistidine, S-Adenosylhomocysteine, beta-D-Glucosamine, and palmitoylethanolamide with the genes were low. In contrast, most of the other DAMs and DEGs exhibited significant or extremely significant positive or negative correlations. The accumulation of these metabolites is primarily influenced by ribose and enzyme genes, suggesting that these genes are directly or indirectly involved in regulating metabolite levels (Figure 13).

#### 3.5.2. Analysis of Key Pathways

**Figure 14:**
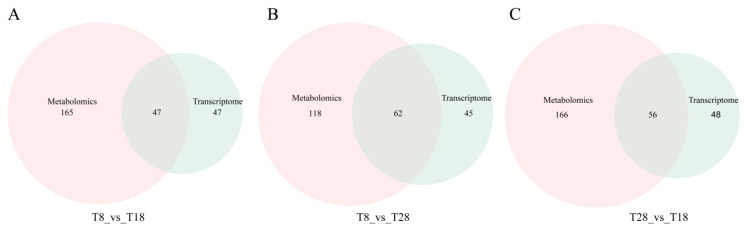
Venn plots of the number of pathways co-enriched by the transcriptome and metabolome in different comparison groups ((**A**) T8 vs. T18, (**B**) T8 vs. T28, (**C**) T28 vs. T18).

**Figure 15:**
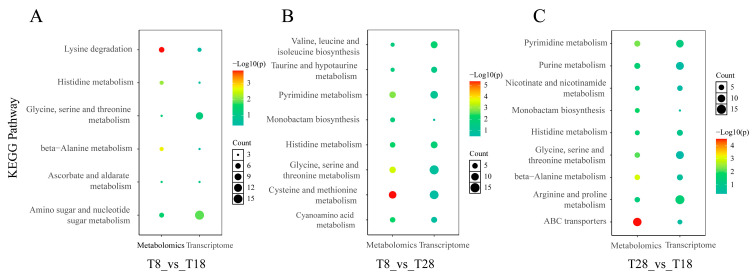
Bubble plots of the KEGG pathway significantly co-enriched by the transcriptome and metabolome in different comparison groups ((**A**) T8 vs. T18, (**B**) T8 vs. T28, (**C**) T28 vs. T18).

To identify the common pathways among the three distinct comparison groups, we mapped the differentially abundant metabolites (DAMs) and differentially expressed genes (DEGs) from these groups to the KEGG database. The analysis revealed that 47, 62, and 56 pathways were co-enriched by DAMs and DEGs in the comparisons of T8 vs. T18, T8 vs. T28, and T28 vs. T18, respectively (Figure 14). Further examination of the common pathways among these groups indicated that DAMs and DEGs were predominantly enriched in lysine degradation, cysteine and methionine metabolism, and ABC transporters (Figure 15). In the lysine degradation pathway, one transcription factor (SPAC1002.12c) was significantly upregulated, while two transcription factors (fap1 and fap2) were significantly downregulated. In the cysteine and methionine metabolic pathways, five transcription factors (eca39, gsa1, SDS, mmuM, and metXA) were significantly upregulated, whereas nine transcription factors (GOT2, cysD, cys-17, serA, metXA, FUB3, xorIIM, swnA, and SPAC56E4.03) were significantly downregulated. In the ABC transporter pathway, one transcription factor (atrD) was significantly upregulated, and three transcription factors (atrD, STE6, and atrC) were significantly downregulated. The significant enrichment of cysteine and methionine metabolites indicates that the strain’s response to varying temperatures may be linked to the accumulation of these metabolites.

### 3.6. qRT-PCR to Verify RNA-Seq Data

**Figure 16:**
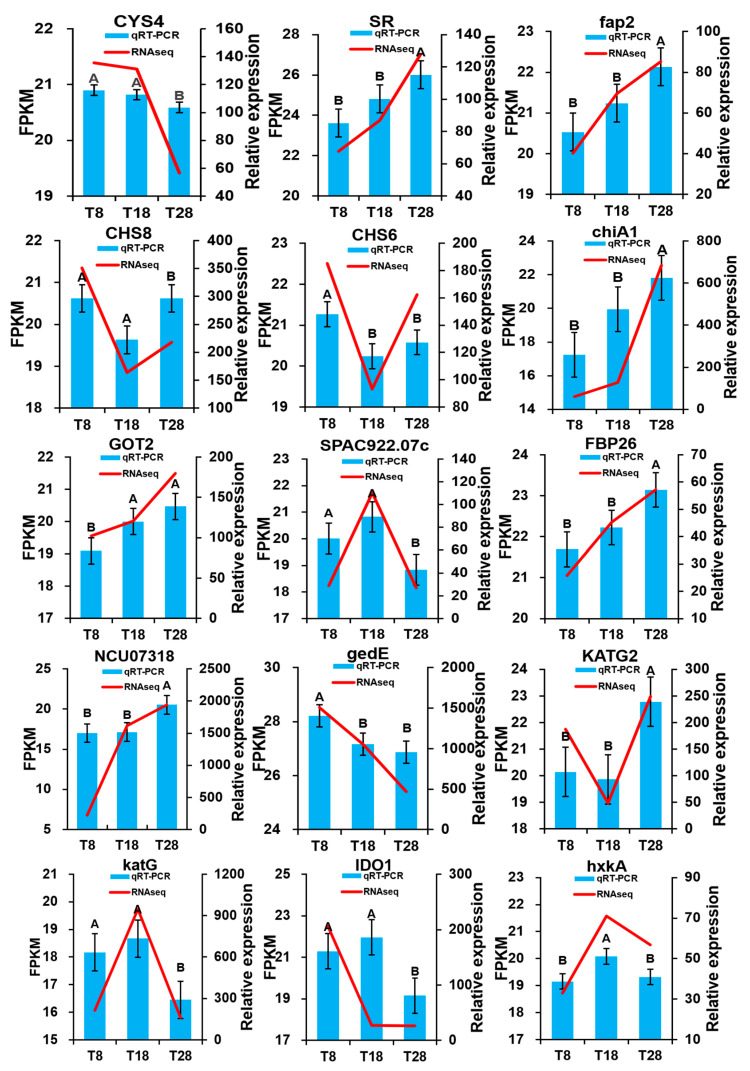
The qRT-PCR validation of 15 DEGs. The different letters under the columns indicate significant differences (*p* < 0.05), and the error bar indicates three biologically repeated means ± SEM.

To verify the stability and reliability of the transcriptome data, we selected 15 differentially expressed genes (DEGs) associated with tryptophan metabolism, arginine and proline metabolism, phenylalanine metabolism, ribosome function, and ribosome biogenesis in eukaryotes. These genes were validated using quantitative reverse transcription polymerase chain reaction (qRT-PCR). The relative expression profiling analysis demonstrated that the expression trends of the 15 DEGs in qRT-PCR and RNA-Seq were similar and exhibited a high correlation (R^2^ ≥ 0.8), indicating that the transcriptomic results were both reliable and accurate (Figure 16).

## 4. Discuss

*O. sinensis* has been widely recognized as a traditional Chinese medicinal herb for the treatment of various diseases. Its notable biological activities, including anti-cancer properties, blood sugar regulation, and immune modulation, have attracted considerable attention and favor from the public [29,30]. However, temperature is a critical factor influencing the growth rate of *O. sinensis*; excessively high or low temperatures can inhibit its growth. Under varying temperature conditions, the enzymatic activity of strain, as well as the synthesis and decomposition of metabolites, undergo significant changes [31]. Therefore, temperature adjustments can impact the metabolic pathways of *O. sinensis*, making the optimization of product quality and yield essential for enhancing its efficiency. This study examined the effects of temperature stress on the growth of *O. sinensis* mycelium. Significant differences were observed in various parameters, including mycelium growth rate, colony surface characteristics, dry and fresh weight, and conidia count, with an optimal temperature range for mycelium growth identified as 18 °C to 23 °C. Additionally, at 28 °C, mycelium growth was monitored over 12 days, revealing an increase in colony diameter of only 0.05 to 0.1 cm, indicating extremely slow growth or even cessation. This suggests that *O. sinensis* is unsuitable for growth at elevated temperatures, which can inhibit mycelial development. Previous studies have indicated that the optimal temperature for fungal colony growth varies, with P. suffultus thriving at 15 °C and C. farinosa and C. coleopterorum preferring 20 °C. Notably, spore germination is most vigorous at 25 °C, whereas temperatures as low as 5 °C completely halt spore germination across all fungal species [32].

Mycelium growth is influenced by various environmental conditions, with temperature being a critical factor. Numerous enzymes are involved in the growth of mycelia; however, these enzymes can only be utilized by the mycelia after undergoing enzymatic decomposition [33]. In an optimal environment, the strain enters the growth phase and initiates conidial germination. The germination and subsequent growth of slender mycelia occur only under suitable temperatures, humidity, and adequate nutritional conditions. At this stage, the mycelia grow at their fastest rate, continuously forming new branches through division and extension. This underscores the close relationship between mycelial growth and environmental factors, as well as nutrient availability [34].

Antioxidant enzymes constitute a class of enzymes that play a crucial role in mitigating oxidative damage in living organisms. They are capable of reducing the production of oxygen free radicals and other ROS, thereby regulating the REDOX balance and protecting cells from damage [35]. Oxidation is a fundamental process that many organisms undergo during energy conversion. Uncontrolled sources of oxygen, including ROS, can be categorized into two main types, namely free radicals and peroxides, both of which can generate free radicals that lead to oxidative damage in organisms. This oxidative damage can further accelerate degenerative processes, such as aging [8,36]. Therefore, the essence of antioxidation lies in delaying or preventing the oxidative degradation of cellular substrates [37]. Reactive oxygen species within organisms typically require various enzymes for decomposition to achieve the effect of delaying aging. In the lower temperature range of 8–13 °C, all three antioxidant enzymes exhibited an increasing trend. This suggests that when the strain detects potential environmental changes due to the gradual rise in temperature, it actively enhances the activity of antioxidant enzymes to manage the anticipated increase in reactive oxygen species (ROS) production. ROS are continuously generated during the metabolic processes of the strain, and excessive accumulation can lead to damage to cellular structures and functions. The increased activity of antioxidant enzymes during this period can effectively eliminate these ROS, thereby maintaining redox balance within the cells. When the temperature ranged from 13 to 23 °C, the activity changes in catalase (CAT), superoxide dismutase (SOD), and peroxidase (POD) exhibited notable differences. CAT and SOD gradually increased their activity within this range, indicating the strain’s strong adaptability to these temperatures. Although POD activity also increased, its magnitude and rate of change diverged from those of the former two enzymes, reflecting the unique response patterns of different antioxidant enzymes as the strain adjusts to temperature changes. The enzymes cooperate with one another while focusing on different aspects, collectively contributing to the formation of an antioxidant defense network. However, when the temperature reaches 28 °C, the activities of catalase (CAT) and superoxide dismutase (SOD) significantly decrease. This decrease indicates that elevated temperatures can disrupt the spatial structure of enzyme proteins, leading to alterations in the active sites of the enzymes, which drastically reduces their catalytic efficiency. Consequently, the strain’s ability to scavenge reactive oxygen species diminishes, resulting in a substantial accumulation of these species within the cells and triggering oxidative stress. This oxidative stress inflicts severe damage on the strain’s cells, compromising the integrity of cell membranes and impairing the functions of organelles, ultimately threatening the normal growth and development of the plant strain. The observed increase in peroxidase (POD) activity suggests that it may possess a more stable protein structure, or that structural changes at elevated temperatures have a lesser impact on its activity, thereby allowing it to maintain a higher level of activity.

The study of metabolomics involves the investigation of endogenous metabolic changes induced by both internal factors within organisms and external environmental stimuli. This field allows for the quantitative and qualitative analysis of key compounds that describe the metabolic cycle, facilitating the exploration of the relationship between metabolites and physiological as well as pathological changes [38,39]. Fungal metabolites possess the ability to counteract various biological and abiotic stresses and play a significant role in human health due to their diverse biological properties [40]. Temperature is one of the most critical factors influencing fungal growth. In this study, we employed metabolomics to analyze and compare the metabolite profiles of *O. sinensis* cultivated under different temperature conditions. The analysis using principal component analysis (PCA) and orthogonal partial least squares discriminant analysis (OPLS-DA) models revealed the differences in metabolites produced by *O. sinensis* under low, moderate, and high temperature cultivation conditions. By comparing metabolites between T8 vs. T18, T8 vs. T28, and T28 vs. T18, we identified 103, 162, and 153 DAMs, respectively. Notably, the number of DAMs in the low-temperature and warm-temperature comparison groups was lower than that in the high-temperature comparison group. The identified DAMs across the three comparison groups predominantly included Threonine, Catechol, L-Prolinamide, Taurine, Creatine, and Acetylphosphate. The change in temperature can affect the activity of enzymes related to threonine anabolism in Cordyceps sinensis and thereby influence the content of acid [41]. L-Prolinamide has been shown to inhibit the release of melanocyte-stimulating hormone (MSH), improve pharmacological mechanisms within the central nervous system, and exhibit antioxidant effects that enhance sperm quality [42]. Taurine has potential mechanisms for regulating the tumor microenvironment and tumor cell metabolism, contributing to enhanced immunotherapy through the modulation of tumor microenvironment metabolism and relevant molecules, thereby exerting anti-tumor effects [43]. The varying levels of these active secondary metabolites in strains at different temperatures suggest that the nutrient composition and medicinal value of the fungi are altered in response to changes in growth environment temperature, indicating that temperature fluctuations may lead to inconsistencies in the metabolite synthesis patterns among strains.

The KEGG databases serve as essential tools for elucidating the advanced functions and applications of cells, organisms, and ecosystems through molecular-level information. Consequently, the KEGG pathway analysis is crucial for comprehending the roles of metabolites and their interrelationships. The KEGG enrichment analysis of differentially accumulated metabolites (DAMs) across three distinct groups revealed that, in the comparisons between low-temperature strains, warm-temperature strains, and high-temperature strains (T8 vs. T18 and T8 vs. T28), the metabolic pathways significantly enriched included lysine degradation, lysine biosynthesis, and protein digestion and absorption. In the comparison between high-temperature and temperate strains (T28 vs. T18), significant enrichment was observed in the metabolism of linoleic acid and cysteine-methionine. Amino acids, recognized for their beneficial effects on human health, play a crucial role in enhancing immunity and promoting wound healing [44]. However, in filamentous fungi, the type of amino acids is a critical factor influencing the growth rate of mycelia [45]. Cysteine functions as an antioxidant and plays a crucial role in the synthesis of antioxidant substances. It effectively scavenges reactive oxygen species generated during metabolism, reduces oxidative damage, enhances the organism’s resistance to oxidative stress, and maintains the stability of cellular structure and function under adverse conditions such as high temperatures and high salinity [46].

With the continuous advancement of transcriptomics, its application has expanded to various fields, including animals, plants, microorganisms, and medicine [47,48]. By investigating organisms through transcriptomic analysis, researchers can identify changes in gene expression across different species [49]. In this study, transcriptomics were employed to examine the differentially expressed genes of *O. sinensis* under varying temperature treatments, leading to the identification of these genes. In the T8 vs. T18 comparison group, the majority of genes were down-regulated. Conversely, in the T8 vs. T28 comparison group, up-regulated genes predominated, while in the T28 vs. T18 comparison group, down-regulated genes outnumbered up-regulated ones. As temperature increased, the number of up-regulated genes gradually decreased, whereas down-regulated genes increased. Additionally, the clustering analysis of the three strains at different temperatures revealed that the strains at T8 and T18 clustered together, whereas those at T28 formed a distinct cluster. Integrating these findings with a comprehensive analysis of morphology, physiological indicators, and metabolomics suggests that the strains at 28 °C may experience high-temperature stress. Ultimately, significant changes were observed in growth rates, physiological and biochemical indices, differential metabolites, and the number of differentially expressed genes. These results indicate that the antioxidant oxidase system of the strain was compromised under different temperature stresses, leading to reduced spore production and adversely affecting normal growth and development. The variability in the number of differentially expressed genes among the strain highlights differing trends in expression. Furthermore, gene expression profiles under varying temperature stresses differed, reflecting the strain’s adaptive mechanisms to temperature fluctuations.

In recent years, the integration of high-throughput sequencing platforms with RNA-Seq technology has significantly advanced molecular biology research [50]. Consequently, this study conducted Gene Ontology (GO) and Kyoto Encyclopedia of Genes and Genomes (KEGG) enrichment analyses of differentially expressed genes (DEGs) across various comparison groups using Illumina RNA-Seq technology. The GO enrichment analysis revealed that DEGs were primarily categorized into three functional domains: molecular function (MF), biological process (BP), and cellular component (CC). Further analysis indicated that the most prominent GO terms in the T8 vs. T18 comparison group included nucleolus, pre-ribosome, 90s ribosome, RNA binding, biological origin of ribosomes, ribosome processing, and metabolic processes. In the T8 vs. T28 comparison group, GO terms were predominantly enriched in extracellular regions and intrinsic components of the plasma membrane. The main GO terms enriched in the T28 vs. T18 comparison group included membrane composition, isotropic transport activity, and solute cation symporter activity. Temperature stress induces the peroxidation of the *O. sinensis* plasma membrane, resulting in compositional changes to adapt to elevated external temperatures [51]. This suggests that temperature stress significantly impacts the metabolic substances of strains, leading to continuous alterations in their physiological, biochemical, and metabolic processes. Additionally, the KEGG pathways associated with DEGs across different comparison groups were analyzed. The results indicated that in the T8 vs. T18 comparison group, DEGs were highly enriched in KEGG pathways related to ribosome biogenesis, as well as the biosynthesis of ubiquinone and other terpenoid quinones in eukaryotes. In the T8 vs. T28 comparison group, DEGs showed high enrichment in KEGG pathways related to protein efflux, tryptophan metabolism, arginine and proline metabolism, protein processing in the endoplasmic reticulum, and phenylalanine metabolism. The KEGG pathways associated with DEGs in the T28 vs. T18 comparison group were significantly enriched in ribosome and eukaryotic ribosome biogenesis pathways. This evidence supports the notion that temperature variations influence the original gene expression patterns of *O. sinensis*, and that the strain’s stress response mechanisms to temperature changes are distinct.

## 5. Conclusions

In this study, we employed multi-omics analysis methods to investigate the changing trends of metabolic pathways in *O. sinensis* under varying cultivation temperature conditions. The variations in temperature influenced the original genetic patterns of *O. sinensis*, and the stress responses and coping mechanisms of the strains exhibited differences when exposed to different temperatures. Furthermore, we elucidated the underlying mechanisms of these variations. Our findings indicate that a culture temperature of 18 °C is optimal for the strains studied. Metabolomic and transcriptomic analyses revealed that elevated temperature conditions predominantly affect the metabolism of linoleic acid, as well as the metabolism of arginine and proline, lysine degradation, and the metabolism of glycine, serine, and threonine. Furthermore, during temperature fluctuations, a significant enrichment of differentially expressed genes (DEGs) and differentially accumulated metabolites (DAMs) that can induce apoptosis and senescence was observed, underscoring their critical roles in influencing growth and development. Temperature stress induces peroxidation of the plasma membrane in *O. sinensis*. To counteract fluctuations in external temperature, the composition of the plasma membrane undergoes alterations, leading to continuous changes in the strain’s physiological, biochemical, and metabolic processes. Furthermore, there are notable differences in the strain’s stress response and response mechanisms to temperature variations. Investigating the optimal culture temperature range for the mycelium, as well as the effects of temperature fluctuations, is crucial for comprehending the internal regulatory mechanisms of *O. sinensis* mycelium. This understanding holds significant implications for industrial development and production.

## Figures and Tables

**Table 1:** Colony surface morphology of strains at different temperatures.

Project	8 °C	13 °C	18 °C	23 °C	28 °C
Surface of colonies	Fluffy and raised	Fluffy and raised	Smooth and fluffy	Smooth and fluffy	Embossment
Texture of colonies	Cottony	Cottony	Cottony	Cottony	Cottony
Colony margins	Ciliform	Ciliform	Ciliform	Ciliform	Ciliform
Color of colonies	White	White	White	White	White
Shapes of colony	Circles and quasi-circles	Circles and quasi-circles	Circles	Circles	Quasi-circles

**Table 2:** Sequencing data quality evaluation table.

Sample	Raw Reads	Clean Reads	Raw Bases (bp)	Clean Bases (bp)	Error Rate (%)	Q20 (%)	Q30 (%)
T8-1	49,131,672	48,135,782	7,418,882,472	7,241,725,861	0.004924	97.39	95.34
T8-2	51,482,918	50,446,216	7,773,920,618	7,583,357,531	0.004884	97.39	95.37
T8-3	48,227,856	47,178,518	7,282,406,256	7,098,646,944	0.004914	97.33	95.24
T18-1	46,194,078	45,264,536	6,975,305,778	6,814,899,717	0.004838	97.40	95.38
T18-2	43,531,594	42,589,292	6,573,270,694	6,407,805,162	0.004874	97.34	95.22
T18-3	45,794,002	44,845,290	6,914,894,302	6,742,696,440	0.004709	97.36	95.32
T28-1	40,729,050	39,890,252	6,150,086,550	5,997,410,785	0.004819	97.43	95.45
T28-2	49,759,690	48,713,274	7,513,713,190	7,324,539,378	0.004916	97.39	95.37
T28-3	50,352,736	49,317,380	7,603,263,136	7,422,575,006	0.004969	97.42	95.41

## Data Availability

The original contributions presented in this study are included in the article. Further inquiries can be directed to the corresponding authors.
